# High-Intensity Interval Training Attenuates Inflammation in Cardiorenal Syndrome Induced by Renal Ischemia–Reperfusion Injury in Rats

**DOI:** 10.3390/life16010044

**Published:** 2025-12-26

**Authors:** Po-Chien Tsao, Chang-Chi Lai, Szu-Kai Fu, Chia-Hsien Yu, Jing-Hsuan Chen, Chia-Yu Tang

**Affiliations:** 1 Graduate Institute of Sports Training, University of Taipei, Taipei 11153, Taiwan; fk1781616@go.utaipei.edu.tw (P.-C.T.);; 2 Department of Exercise and Health Sciences, University of Taipei, Taipei 11153, Taiwan

**Keywords:** acute kidney injury, ischemia–reperfusion injury, high-intensity interval training, myocardial injury

## Abstract

Acute kidney injury (AKI), a common complication of renal and cardiovascular procedures, induces systemic inflammation that can lead to secondary cardiac injury. This study examined whether prior high-intensity interval training (HIIT) could modulate biochemical and histological markers of cardiac injury following renal ischemia–reperfusion (I/R). Thirty male Sprague–Dawley rats were assigned to Sham, Renal I/R, and HIIT groups; one rat in the I/R group and two in the HIIT group did not survive. Serum analyses included creatinine, CK, troponin I, LDH, and inflammatory cytokines. Renal injury was assessed using tubular and glomerular injury scores, and cardiac injury was evaluated by myocardial injury scoring, TUNEL staining, and expression of caspase-3, TNF-α, and Bax. Renal I/R induced renal dysfunction, systemic inflammation, and myocardial damage. Prior HIIT significantly reduced creatinine, CK, troponin I, LDH, inflammatory cytokines, and cardiac caspase-3 and TNF-α expression. Overall, HIIT provided partial protection against renal I/R-induced systemic and cardiac alterations, primarily by attenuating inflammation, and should be considered a potential—rather than definitive—preconditioning strategy requiring further investigation.

## 1. Introduction

Ischemia–reperfusion (I/R) occurs when blood supply to a tissue is completely interrupted, leading to ischemia and hypoxia. Restoration of blood flow is essential for the recovery of ischemic tissue [1,2]. However, paradoxically, it may also induce further injury. I/R is therefore recognized as a major cause of morbidity and mortality in conditions such as stroke, cardiovascular ischemia, and complications related to organ transplantation [3]. This occurs because the reperfused blood can damage the previously ischemic organ, thereby compromising its function and viability. The resulting pathology, termed I/R injury, may extend beyond the affected tissue to trigger systemic responses, injuring distant organs and potentially progressing to multiple organ failure [1,4,5].

The kidney is especially susceptible to I/R injury because of its distinctive structural and functional features, most notably its high oxygen demand [6]. Renal IRI frequently arises in sepsis, kidney transplantation, and cardiac surgery, and often culminates in acute kidney injury (AKI) [3,7]. AKI can subsequently progress to chronic kidney disease or even end-stage kidney disease [8], with a reported mortality rate of approximately 23% [9]. Most AKI-related deaths stem not from renal failure itself but from cardiac complications or sepsis [10,11]. Cardiovascular disease remains the predominant complication of chronic kidney disease [12], and renal interventions have been strongly associated with adverse cardiac outcomes such as myocardial infarction, heart failure, and arrhythmias [13]. These observations underscore the intricate bidirectional relationship between the kidney and the heart, ultimately contributing to cardiorenal syndrome, i.e., combined dysfunction in both organs [13,14,15]. Of the more than 300 million surgeries conducted worldwide annually, roughly 1% involve the kidneys [16], during which renal I/R is often unavoidable. Consequently, strategies that mitigate AKI-induced complications—particularly those affecting the heart—are urgently needed to improve outcomes and reduce mortality.

Since the seminal report by Morris and Crawford in 1958 on the cardioprotective effects of physical activity [17], exercise has been widely recognized for its role in improving functional capacity and reducing the incidence and mortality of myocardial infarction and other cardiovascular conditions [18]. In clinical settings, structured exercise programs enhance patients’ physical performance and cardiorespiratory fitness, thereby reducing hospital admissions and improving quality of life. Moreover, such programs alleviate symptoms in cardiomyopathy and heart failure [19]. Regular exercise can enlarge left ventricular dimensions, increase myocardial wall thickness, augment cardiac output and stroke volume, and simultaneously reduce resting heart rate and infarct size. Collectively, evidence suggests that exercise functions as a non-pharmacological preconditioning strategy to protect the myocardium from I/R injury [20,21,22].

The cardioprotective benefits of exercise depend strongly on its mode, intensity, and duration. Exercise performed at 75–80% of maximal oxygen uptake (VO_2_max), including moderate-to-high-intensity aerobic interval training [23], enhances heart rate and blood flow [24,25,26], induces physiological cardiac hypertrophy [27,28,29,30,31], lowers the production of pro-inflammatory cytokines such as tumor necrosis factor alpha (TNF-α) and (IL)-6, and elevates anti-inflammatory cytokines such as IL-10 [32,33,34]. HIIT has been shown to produce superior improvements in vascular endothelial function and arterial stiffness compared to moderate-intensity continuous training, largely through shear stress-induced increases in nitric oxide bioavailability [35]. These adaptations are further supported by the broader cardiovascular mechanisms described in the literature, including enhanced endothelial signaling, improved mitochondrial efficiency, and reduced oxidative stress [36]. Furthermore, exercise attenuates caspase-3 activation and reduces cardiomyocyte apoptosis [20,37], thereby exerting potent cardioprotective effects. However, most previous studies have investigated these benefits only in the context of cardiac I/R. To date, whether exercise can protect the heart from injury secondary to renal I/R has not been addressed. Therefore, in this study, we aimed to determine whether exercise preconditioning provides cardioprotection against renal I/R-induced cardiac injury and to elucidate the underlying mechanisms.

## 2. Materials and Methods

### 2.1. Animals Preparation and Protocol

This study was approved by the Institutional Animal Care and Use Committee of the University of Taipei, Taiwan (Approval No. UT110004). Two-week-old male Sprague–Dawley rats were obtained from BioLASCO Taiwan Co., Ltd. (Taipei, Taiwan). All experimental procedures complied with the Guide for the Care and Use of Laboratory Animals [38]. Animals were maintained under controlled environmental conditions with a 12 h light/dark cycle. Each rat was housed individually in a plastic enclosure (482 mm × 258 mm × 225 mm) that provided adequate space for movement, with free access to food and water at room temperature.

At 3 weeks of age, 30 male Sprague–Dawley rats were randomly assigned in equal numbers to three groups: Sham, renal I/R, and HIIT. From 3 to 9 weeks of age, rats in the Sham and renal I/R groups were kept under standard housing conditions without exercise intervention, whereas rats in the HIIT group underwent 2 weeks of adaptation training followed by 4 weeks of HIIT, totaling 6 weeks. At 9 weeks of age, rats in the Sham group underwent an abdominal incision without arterial occlusion, while those in the renal I/R and HIIT groups (n = 20 in total) were subjected to renal I/R surgery consisting of 60 min of bilateral renal artery occlusion followed by 24 h of reperfusion.

**Figure 1:**
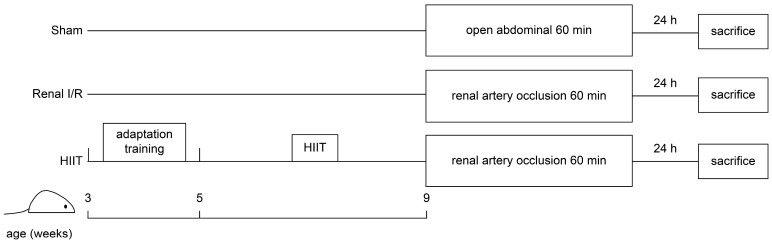
Experimental design. Thirty male Sprague–Dawley rats were randomly assigned to the Sham, renal ischemia–reperfusion (I/R), and high-intensity interval training (HIIT) groups at 3 weeks of age. Rats in the HIIT group underwent 2 weeks of adaptation training followed by 4 weeks of HIIT before renal I/R surgery. All rats were sacrificed 24 h after the surgical procedure.

Blood pressure and heart rate were measured at five time points: pre-surgery (baseline 1), post-abdominal incision (baseline 2), 60 min after renal I/R, and at 3 and 24 h post-surgery (prior to sacrifice). Measurements were obtained using a non-invasive blood pressure system for rats and mice (BP-2010A; Softron Biotechnology, Beijing, China), which employed a specialized tail cuff and pulse sensor to assess tail blood flow while minimizing stress [39]. Rats were euthanized 24 h after the surgical procedure and after collecting blood samples from all animals, and cardiac and renal tissues were immediately harvested for further analysis. A schematic overview of the experimental design is presented in Figure 1.

### 2.2. HIIT Intervention

The HIIT protocol was performed on a T510 treadmill (Diagnostic & Research Instruments, Singa, Taoyuan, Taiwan; Figure S1). Rats assigned to the HIIT group first underwent a 2-week adaptation phase beginning at postnatal week 3. During this period, the treadmill was set at 20 m/min with a 5° incline, and the rats ran for 30 min daily. At postnatal week 5, a 4-week formal HIIT program commenced. Each session began with a warm-up at 15 m/min on a 5° incline, followed by high-intensity bouts at 50 m/min on a 10° incline. The protocol consisted of ten 1 min running intervals interspersed with 2 min rest periods, for a total of 30 min per session, designed to reach ~85% of VO_2_max [40]. This estimated intensity was derived from previously validated treadmill-running VO_2_max calibration studies in juvenile rats, as the present study did not directly measure VO_2_max. Training was conducted four days a week. A mild electrical stimulus (30 V) was delivered only when a rat drifted toward the rear of the treadmill; the stimulus had no fixed duration and occurred infrequently, serving solely as a minimal behavioral cue. Details of the protocol are provided in Table 1. After completing the 6-week HIIT program, rats underwent renal I/R surgery at 9 weeks of age.

### 2.3. Renal I/R

At postnatal week 9, all rats underwent surgery after a 24 h fasting period. Anesthesia was induced with intraperitoneal pentobarbital (30 mg/kg). Animals were placed on a thermoelectric warming pad to maintain body temperature at 37 °C and monitored continuously with a rectal thermometer. The heating device was programmed to shut off if the temperature exceeded 37.5 °C.

A midline abdominal incision was made, and the bilateral renal arteries were occluded for 60 min using nontraumatic microvascular clamps. Successful ischemia was confirmed based on a visible color change in the kidneys. The clamps were released to initiate reperfusion. Throughout the ischemic period, anesthesia was maintained. The abdominal incision was kept open with atraumatic clamps and covered with sterile gauze to preserve asepsis. Following reperfusion, the incision was closed in layers using 4-0 silk sutures. Postoperatively, rats were returned to cages for natural recovery without analgesics or antibiotics. Sham-operated rats underwent identical surgical procedures except for the renal artery occlusion.

### 2.4. Biochemical Study

Blood samples were collected and centrifuged for serum analysis. Biomarkers were quantified using an automated analyzer with colorimetric methods. Assessment of serum biomarkers was carried out via enzyme-linked immunosorbent assay (ELISA) using commercial kits purchased from DRG International, Inc. (Springfield, NJ, USA), according to the manufacturer’s instructions. The parameters measured included blood urea nitrogen (BUN), creatinine, creatine phosphokinase (CPK), lactate dehydrogenase (LDH), and cardiac troponin I (cTnI), which were expressed in the following units: BUN and creatinine in mg/dL, CPK and LDH in U/L, and cTnI in ng/mL.

For biochemical measurements, serum samples were submitted to an independent certified laboratory and labeled only with randomized numeric codes, ensuring complete blinding of laboratory personnel.

### 2.5. Histological Examination

At 24 h post-reperfusion, the abdominal cavity and carotid artery were exposed. Catheters were inserted into the carotid artery and inferior vena cava to perfuse 50 mL phosphate-buffered saline through each vessel, flushing residual blood. The heart and kidneys were excised and fixed in 10% neutral buffered formalin.

The left ventricle was dehydrated, embedded, and sectioned into 5-μm paraffin slices mounted on glass slides. Sections were deparaffinized with xylene and stained with hematoxylin and eosin. Cardiac and renal damage were evaluated by light microscopy.

Cardiac injury was graded on a 0–4 morphological scale: 0, no injury; 1, mild injury with interstitial edema and focal necrosis; 2, moderate injury with diffuse myocardial swelling and necrosis; 3, severe injury with necrosis, contraction bands, and neutrophil infiltration; and 4, extensive necrosis with contraction bands, neutrophil infiltration, and hemorrhage [41]. To obtain a representative injury score for each animal, three H&E-stained sections from the left ventricle were evaluated, and five randomly selected high-power fields per section (400× magnification) were analyzed using an inverted light microscope (CKX53; Olympus Corporation, Tokyo, Japan). For each field, the morphological score (0–4) was assigned based on the predominant injury pattern. The final myocardial injury score for each rat was calculated as the mean score across all the analyzed fields, providing a quantitative estimate of the overall myocardial damage. All scoring was performed in a blinded manner by an experienced evaluator who was unaware of group allocation.

Renal injury was assessed using a four-point scale: 0, no changes; 1, mild localized changes (<25% of the field); 2, moderate multifocal changes (25–50%); and 3, marked widespread changes (>50%). The evaluated features included focal glomerular necrosis, capsular dilation, tubular epithelial degeneration or necrosis, tubular dilation, and interstitial inflammation [42]. To ensure consistency, kidney sections were examined at 400× magnification in ten randomly selected fields by an experienced pathologist who was blinded to the group allocation. The final score for each animal was calculated as the average of all evaluated fields.

### 2.6. Terminal Deoxynucleotidyl Transferase dUTP Nick-End Labeling (TUNEL) Assay

Cardiomyocyte apoptosis was assessed using a TUNEL assay (Roche, Basel, Switzerland) according to the manufacturer’s protocol. Briefly, 3–4 μm heart sections were deparaffinized and treated with proteinase K (20 μg/mL) at 22 °C for 15 min. Sections were incubated with 3% hydrogen peroxide, rinsed in phosphate-buffered saline blocking buffer, and permeabilized. Terminal deoxynucleotidyl transferase was then applied at room temperature for 1 h. The reaction was stopped with wash buffer, and slides were incubated with peroxidase-conjugated anti-digoxigenin antibody for 30 min. Diaminobenzidine (Sigma, St. Louis, MO, USA) was used for chromogenic detection.

Apoptosis was evaluated in a blinded manner by light microscopy. Three sections per left ventricle were analyzed, and 6–8 random regions per section were selected. The percentage of TUNEL-positive nuclei relative to the total number of nuclei was calculated, and only nuclei showing a distinct brown/dark intranuclear TUNEL signal were counted as positive.

### 2.7. Enzyme-Linked Immunosorbent (ELISA) Analysis

Serum cytokines TNF-α, IL-1β, IL-6, and IL-10 were quantified using Quantikine^®^ ELISA kits (R&D Systems, Minneapolis, MN, USA) according to the manufacturer’s protocols.

### 2.8. Western Blotting Analysis

Left ventricular tissue was collected individually from each rat (n = 4 per group), and no pooled samples were used. Each sample was analyzed once per animal. Protein expression of activated caspase-3, TNF-α, and Bax in left ventricular tissue was assessed by Western blotting. Tissues were homogenized in buffer at 4 °C, centrifuged at 12,000× *g* for 10 min, and supernatants collected. Protein concentration was determined using a bicinchoninic acid assay.

Equal amounts of protein were separated on 15% sodium dodecyl sulfate–polyacrylamide gel electrophoresis (SDS-PAGE) gels and transferred to nitrocellulose membranes. Membranes were blocked in TBST containing 0.1% Tween 20 and 5% nonfat milk for 1 h, washed, and incubated overnight at 4 °C with primary antibodies (1:500 for caspase-3; 1:1000 for TNF-α and Bax). After washing, membranes were incubated with appropriate secondary antibodies at 37 °C for 1 h. Protein bands were visualized with chemiluminescent substrate (Immobilon, Merck Millipore, Billerica, MA, USA) and quantified using NIH Image 1.6. Protein levels were normalized to those of β-actin (1:2000 dilution) as a loading control.

Western blot samples were labeled with randomized numeric codes by an investigator not involved in the protein analysis. The researcher performing the Western blotting and densitometry was blinded to group allocation until all analyses were completed.

### 2.9. Statistical Analysis

Data were analyzed using SPSS 22.0 (San Diego, CA, USA) and are expressed as mean ± SEM. Group comparisons were performed using the Mann–Whitney U test or Wilcoxon rank-sum test. A *p*-value < 0.05 was considered significant. Due to perioperative mortality, the final sample size varied across analyses, and outliers were examined and removed using Dixon’s Q test when appropriate. The Benjamini–Hochberg correction was applied to control the false discovery rate. The exact n for each biochemical, histological, TUNEL, and Western blot analysis is reported in the Results and figure legends.

## 3. Results

### 3.1. Mortality and Hemodynamic Changes

Each group initially included 10 rats. In the renal I/R group, one rat died the day after surgery. In the HIIT group, one rat died the following day and another succumbed to fatal intraoperative bleeding (Table 2). Systolic blood pressure and heart rate were comparable across groups at baselines 1 and 2 (Table 3). After 60 min of ischemia, both the renal I/R and HIIT groups exhibited a marked reduction in systolic blood pressure and heart rate. Partial recovery was observed at 3 h of reperfusion; however, by 24 h, both groups again demonstrated significant declines, accompanied by physical debilitation. Compared with the renal I/R group, the HIIT group showed a less pronounced reduction in these parameters at RAO (1 h), RAR (3 h), and RAR (24 h). In contrast, the sham group maintained stable values across all time points.

### 3.2. Analysis of Renal Injury

**Figure 2:**
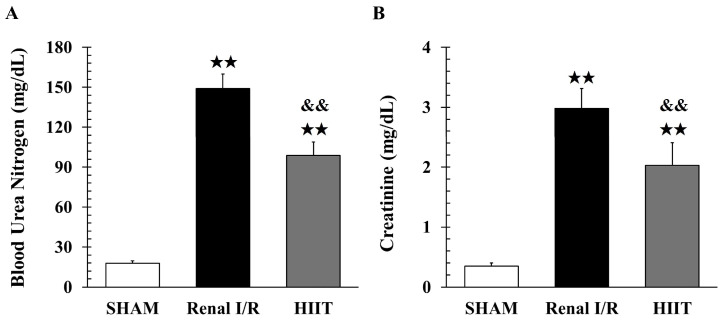
Effects of HIIT on renal function. Renal ischemia–reperfusion (I/R) significantly increased (**A**) blood urea nitrogen (BUN) and (**B**) creatinine levels compared with the sham group, whereas HIIT reduced these elevations. Data are presented as mean ± SD (n = 6 per group). ^★★^ *p* < 0.01 vs. SHAM; ^&&^ *p* < 0.01 vs. Renal I/R. I/R, ischemia–reperfusion; HIIT, high-intensity interval training.

Renal function was assessed in six rats per group, and kidney tissues from four rats per group were examined histologically. A representative appearance of the kidneys during the ischemic period is shown in Figure S2, illustrating the color change from a normally perfused kidney before clamping to a darker, congested appearance after 1 h of ischemia. Additional procedural details during the 60 min ischemia period are shown in Figure S3. Renal I/R markedly increased BUN (149 ± 10.99 vs. 17.83 ± 1.86 mg/dL in sham; adj. *p* = 0.002; Figure 2A) and creatinine (2.98 ± 0.33 vs. 0.35 ± 0.05 mg/dL in sham; adj. *p* = 0.003; Figure 2B). HIIT preconditioning significantly mitigated renal dysfunction induced by renal I/R, as evidenced by lower BUN (98.82 ± 10.13 vs. 149 ± 10.99 mg/dL, adj. *p* = 0.002) and creatinine levels (2.03 ± 0.38 vs. 2.98 ± 0.33 mg/dL, adj. *p* = 0.004).

**Figure 3:**
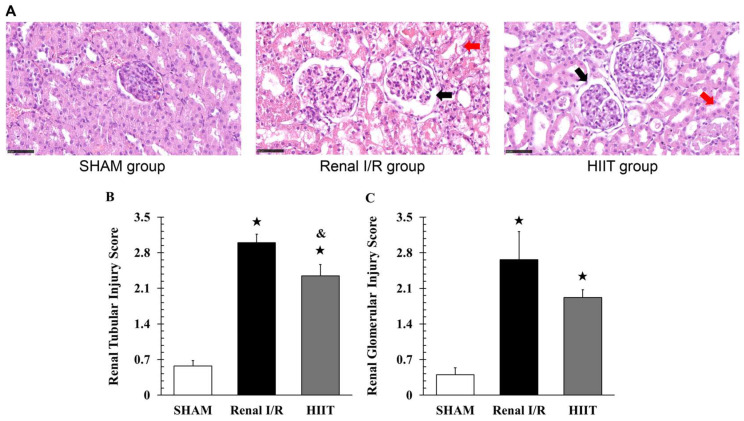
(**A**) Representative H&E-stained kidney sections (400× magnification). SHAM kidneys show normal glomeruli and tubules. Renal I/R kidneys show glomerular dilation and structural disruption (black arrows), and tubular necrosis/dilation (red arrows). HIIT kidneys showed partial improvement in tubular morphology (red arrows), whereas glomerular abnormalities persisted. (**B**,**C**) Quantitative tubular and glomerular injury scores. Data are presented as mean ± SD (n = 4). ^★^ *p* < 0.05 vs. SHAM; ^&^ *p* < 0.05 vs. renal I/R (tubular score only). I/R, ischemia–reperfusion; HIIT, high-intensity interval training.

Histological evaluation revealed normal glomeruli and tubules in the sham group (Figure 3A). In contrast, renal I/R produced marked degeneration, necrosis, and mild dilation of glomeruli and tubules. Injury scores were significantly higher in the renal I/R than in the sham group (tubules: 3.00 ± 0.17 vs. 0.57 ± 0.11, adj. *p* = 0.044; glomeruli: 2.67 ± 0.56 vs. 0.40 ± 0.14, adj. *p* = 0.029; Figure 3B,C). HIIT preconditioning attenuated renal injury (tubules: 2.35 ± 0.23 vs. 3.00 ± 0.17, adj. *p* = 0.029; glomeruli: 1.92 ± 0.15 vs. 2.67 ± 0.56, adj. *p* = 0.114; vs. renal I/R). Together, the results suggest that renal I/R significantly impaired renal function and structure, whereas prior HIIT conferred protective effects.

### 3.3. Biochemical Analysis of Myocardial Injury

**Figure 4:**
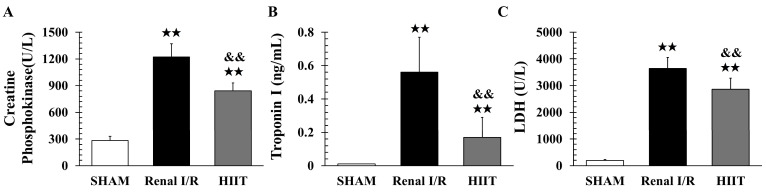
Serum markers of myocardial injury. Levels of (**A**) creatine phosphokinase (CPK), (**B**) cardiac troponin I (cTnI), and (**C**) lactate dehydrogenase (LDH) were significantly elevated in the renal ischemia–reperfusion (I/R) group compared with the sham group. HIIT reduced these elevations. Data are presented as mean ± SD (n = 6 per group). ^★★^ *p* < 0.01 vs. SHAM; ^&&^ *p* < 0.01 vs. renal I/R. I/R, ischemia–reperfusion; HIIT, high-intensity interval training.

Serum markers of cardiac injury (CPK, LDH, and cTnI) were assessed in six rats per group. Compared with sham, the renal I/R group exhibited significant elevations in CPK (1222.17 ± 147.07 vs. 282.17 ± 47.60 U/L; adj. *p* = 0.002; Figure 4A), cTnI (0.56 ± 0.21 vs. 0.01 ± 0.00 ng/mL; adj. *p* = 0.003; Figure 4B), and LDH (3635.83 ± 421.38 vs. 197 ± 39.13 U/L; adj. *p* = 0.003; Figure 4C). HIIT preconditioning significantly attenuated these increases (CPK: 842.17 ± 88.15 vs. 1222.17 ± 147.07 U/L, adj. *p* = 0.002; cTnI: 0.17 ± 0.12 vs. 0.56 ± 0.21 ng/mL, adj. *p* = 0.004; LDH: 2858 ± 421.41 vs. 3635.83 ± 421.38 U/L, adj. *p* = 0.015).

### 3.4. Histological Examination of Myocardial Injury

**Figure 5:**
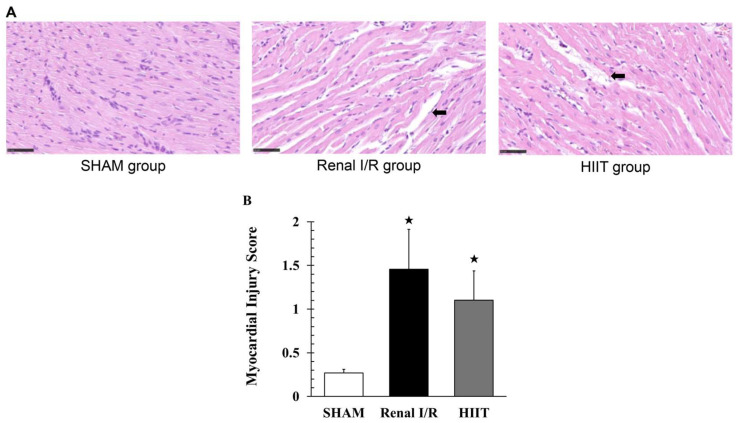
(**A**) Representative H&E-stained myocardial sections (400×) from the SHAM, renal ischemia–reperfusion (I/R), and HIIT groups. SHAM myocardium shows normal, well-aligned fibers without edema or disruption. Renal I/R myocardium exhibits marked interstitial edema and waviness/disruption of cardiomyocyte fibers (black arrow). HIIT myocardium shows mild interstitial edema and fiber disarray, qualitatively less pronounced than in the renal I/R group (black arrow). (**B**) Quantification of myocardial injury scores. Data are presented as mean ± SD (n = 4 per group). ^★^ *p* < 0.05 vs. SHAM. I/R, ischemia–reperfusion; HIIT, high-intensity interval training.

Hematoxylin and eosin staining of the left ventricle was performed in four rats per group. Sham animals displayed normal myocardial morphology without interstitial edema or necrosis (Figure 5A). Renal I/R induced mild to moderate interstitial edema, cardiomyocyte swelling, and fiber disruption (scores 1–2). Myocardial injury scores were significantly increased in the renal I/R group compared with sham (1.457 ± 0.455 vs. 0.268 ± 0.042; adj. *p* = 0.044; Figure 5B). In the HIIT group, myocardial sections showed milder interstitial edema and less pronounced fiber waviness compared with the renal I/R group, although localized areas of fiber disarray were still present. These qualitative improvements indicate partial but incomplete structural protection. HIIT preconditioning alleviated these histological changes, showing a non-significant reduction compared with renal I/R (**adj.** *p* = 0.20), but scores remained significantly higher than sham (adj. *p* = 0.044).

### 3.5. TUNEL Staining Analysis of the Myocardium

**Figure 6:**
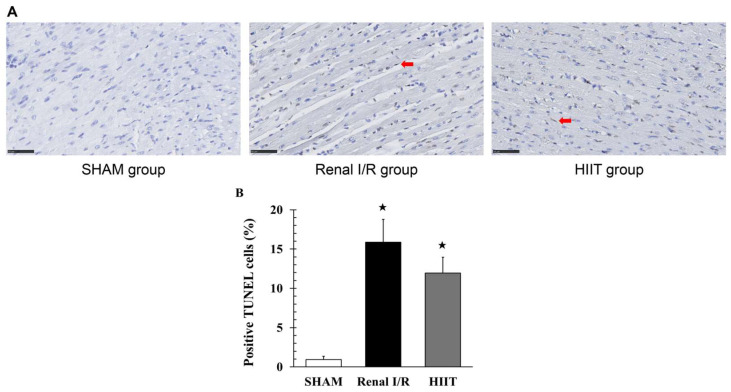
(**A**) Representative TUNEL-stained myocardial sections (400× magnification). SHAM myocardium shows almost no TUNEL-positive nuclei. Renal I/R myocardium exhibits numerous TUNEL-positive apoptotic nuclei (red arrows). HIIT myocardium shows multiple TUNEL-positive nuclei, although qualitatively fewer than those observed in the renal I/R group (red arrows). (**B**) Quantification of TUNEL-positive cells. Data are presented as mean ± SD (n = 4). ^★^ *p* < 0.05 vs. SHAM. I/R = ischemia–reperfusion; HIIT = high-intensity interval training.

TUNEL staining was performed on left ventricles from four rats per group. No apoptotic changes were detected in sham (Figure 6A). Renal I/R significantly increased TUNEL-positive cardiomyocytes (15.87 ± 2.89% vs. 0.94 ± 0.39% in sham; adj. *p* = 0.044; Figure 6B). HIIT reduced the proportion of apoptotic cells (11.94 ± 2.02% vs. 15.87 ± 2.89% in renal I/R), but the reduction was not significant (adj. *p* = 0.114), although it did show a downward trend.

### 3.6. Analysis of Inflammatory Factors

**Figure 7:**
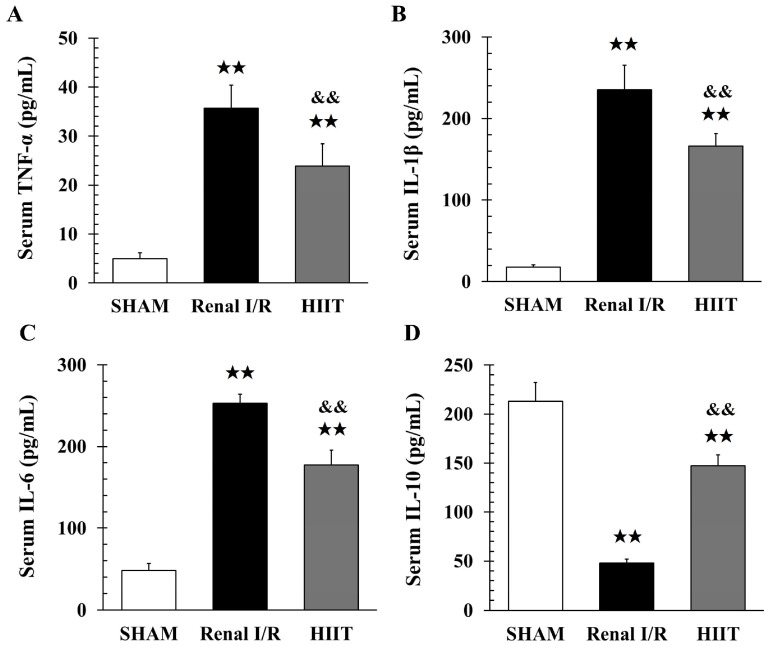
Serum cytokine levels measured by ELISA. Renal ischemia–reperfusion (I/R) markedly increased serum (**A**) TNF-α, (**B**) IL-1β, and (**C**) IL-6 levels compared with the sham group, while (**D**) IL-10 levels were significantly reduced. HIIT significantly attenuated the elevations of TNF-α, IL-1β, and IL-6, and partially restored IL-10 levels compared with the renal I/R group. Data are presented as mean ± SD (n = 6 per group). ^★★^ *p* < 0.01 vs. SHAM; ^&&^ *p* < 0.01 vs. renal I/R. I/R, ischemia–reperfusion; HIIT, high-intensity interval training.

Systemic cytokine levels were quantified using ELISA. Renal I/R markedly increased serum TNF-α (35.70 ± 4.6 vs. 4.93 ± 1.2 pg/mL in sham; **adj.** *p* = 0.002; Figure 7A), IL-1β (235.2 ± 30.1 vs. 17.74 ± 2.8 pg/mL; **adj.** *p* = 0.002; Figure 7B), and IL-6 (253.05 ± 10.8 vs. 48.24 ± 8.5 pg/mL; **adj.** *p* = 0.002; Figure 7C). HIIT significantly attenuated these elevations (TNF-α: 23.92 ± 4.4, **adj.** *p* = 0.002; IL-1β: 166.1 ± 15.4, **adj.** *p* = 0.002; IL-6: 177.1 ± 18.4 pg/mL, **adj.** *p* = 0.002 vs. renal I/R).

IL-10 levels were markedly reduced in the renal I/R group (47.65 ± 4.3 vs. 213.2 ± 18.8 pg/mL in sham; **adj.** *p* = 0.002; Figure 7D). Although HIIT did not restore IL-10 to sham levels, it significantly increased IL-10 compared with renal I/R (147.1 ± 11.3 vs. 47.6 ± 4.3 pg/mL; **adj.** *p* = 0.002).

### 3.7. Cardiac Western Blot Analysis

**Figure 8:**
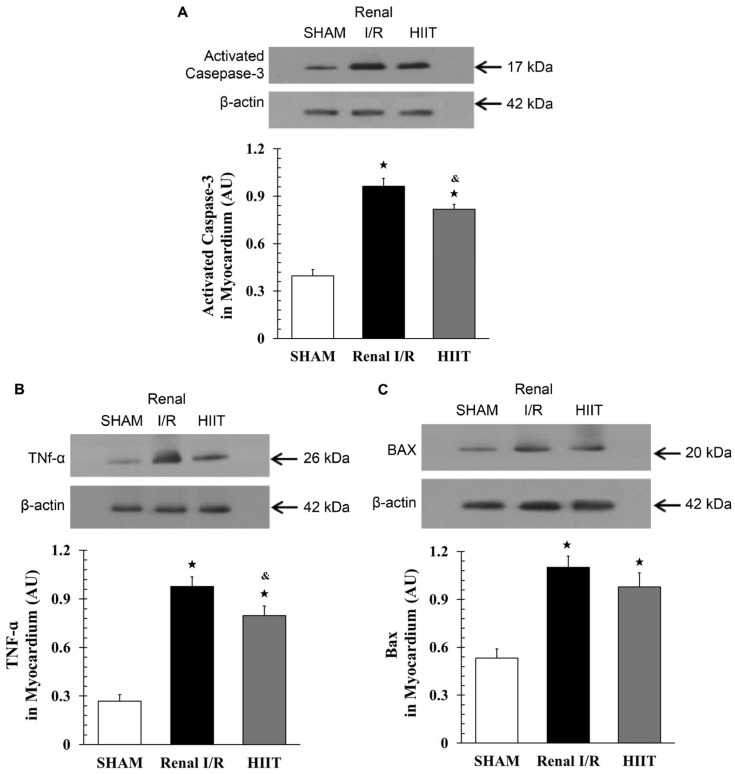
Western blot analysis of myocardial proteins. Representative blots and quantification of (**A**) activated caspase-3, (**B**) TNF-α, and (**C**) Bax expression in the myocardium. Protein levels were normalized to that of β-actin. Renal ischemia–reperfusion (I/R) significantly increased caspase-3, TNF-α, and Bax levels compared with the sham group. HIIT reduced caspase-3 and TNF-α expression and showed a slight, non-significant reduction in Bax. Data are presented as mean ± SD (n = 4 per group). ^★^ *p* < 0.05 vs. SHAM. ^&^ *p* < 0.05 vs. renal I/R. I/R, ischemia–reperfusion; HIIT, high-intensity interval training.

Western blot analysis was performed on myocardial tissue from four rats per group. Renal I/R markedly increased activated caspase-3 levels compared to sham (lane 2: 96.35 ± 5.05 vs. 39.69 ± 3.91; adj. *p* = 0.029; Figure 8A). HIIT significantly attenuated this increase, although levels did not fully return to sham values (lane 3: 81.76 ± 3.00 vs. 96.35 ± 5.05 in renal I/R; adj. *p* = 0.029; Figure 8A). Similarly, myocardial TNF-α expression was lowest in sham (lane 1) and significantly increased by renal I/R (lane 2: 4.94 ± 1.25 vs. 35.71 ± 4.67; adj. *p* = 0.002; Figure 8B). HIIT preconditioning prevented this elevation (lane 3: 23.93 ± 4.47 vs. 35.71 ± 4.67 in renal I/R; adj. *p* = 0.002).

The expression of Bax, a pro-apoptotic protein upstream of caspase-3 activation, was also elevated by renal I/R compared with sham (lane 2: 110.24 ± 6.87 vs. 53.25 ± 5.69; adj. *p* = 0.029; Figure 8C). HIIT slightly reduced Bax levels, although the change was not significant compared with that following renal I/R (97.87 ± 8.80 vs. 110.24 ± 6.87; adj. *p* = 0.114). Collectively, these findings indicate that renal I/R upregulated myocardial pro-inflammatory and pro-apoptotic proteins, whereas HIIT preconditioning suppressed caspase-3 and TNF-α expression, with Bax showing a partial reduction.

## 4. Discussion

Evidence increasingly highlights the strong association between AKI and cardiovascular disease. Regardless of AKI severity, the risk of cardiovascular events, including heart failure, increases substantially within the first year after AKI onset [43]. Compared with patients without AKI, those with AKI have a relative risk of 1.38 for cardiovascular events and 1.86 for cardiovascular mortality [44]. Thus, in patients with AKI, mortality is often driven not by kidney injury alone but by secondary cardiac injury. AKI induces systemic inflammation that propagates intrinsic changes in distant organs, particularly the heart [45]. Consequently, preventing and mitigating the cardiovascular sequelae of AKI has become a key research priority. Exercise, which improves vascular function across different populations, has been consistently associated with reduced overall mortality [44,45,46,47,48,49,50]. In this context, our study provides preliminary experimental evidence that HIIT attenuates systemic and myocardial inflammation in a rat model of AKI. Myocardial effects appear to be limited and primarily related to inflammatory modulation rather than structural myocardial changes.

The AKI animal model that we used in this study was adapted from previously established protocols [51,52]. We selected this bilateral renal ischemia–reperfusion model because it is a well-established and widely used experimental approach that reliably produces acute kidney injury and remote cardiac injury, making it suitable for studying cardiorenal interactions. Bilateral renal arteries were occluded with microvascular clamps for 60 min, followed by 24 h of reperfusion. This procedure effectively mimicked the clinical presentation of AKI, as demonstrated by elevated serum creatinine and BUN levels [53]. Histological findings confirmed glomerular and tubular degeneration and necrosis, consistent with ischemic injury and reflective of AKI severity. Hemodynamic parameters showed a decreasing trend, whereas serum myocardial injury markers (CPK, LDH, and cTnI) were markedly elevated. Furthermore, the left ventricle showed structural disruption, including fiber rupture, interstitial edema, cardiomyocyte swelling, and apoptosis, confirming myocardial injury secondary to renal I/R. Prem and Kurian [54] reported that in Wistar rats, 45 min of bilateral renal artery clamping followed by 24 h of reperfusion did not significantly impair cardiac function, whereas reperfusion extended to 48 h produced significant hemodynamic decline. Such discrepancies underscore the model-dependent nature of ischemic AKI pathology, which varies with species, strain, sex, age, ischemia duration, and surgical conditions [55,56,57,58]. In our study, additional factors, such as clamping renal pedicles rather than arteries, a larger abdominal incision, and an abdominal (versus dorsal) approach, may have exacerbated AKI severity, thereby amplifying myocardial damage. Taken together, our results support the utility of this rat model for studying cardiorenal interactions mediated by renal I/R.

HIIT has been shown to reduce myocardial infarction size and protect the heart from I/R injury [59,60,61,62,63,64], but its role in renal I/R-induced cardiac injury has not been previously explored. In this study, we demonstrated that HIIT significantly improved several AKI-related outcomes. Specifically, blood urea nitrogen (BUN) and creatinine levels, tubular injury scores, cardiac troponin I, inflammatory cytokines, and myocardial expression of caspase-3 and TNF-α were all significantly reduced in HIIT-treated rats compared those to in the renal I/R group. Other parameters, including Bax expression, glomerular injury, H&E-stained myocardial morphology, and TUNEL-positive cardiomyocytes, showed modest decreases relative to the renal I/R group, although these changes did not reach statistical significance. Taken together, these findings indicate that HIIT confers partial cardioprotective and renoprotective effects, primarily reflected in the variables that showed significant improvement. Exercise reduces apoptosis, promotes mitochondrial biogenesis, and enhances myocardial energy metabolism [65], thereby improving cardiac resilience to I/R. Unlike pathological hypertrophy, exercise-induced physiological hypertrophy represents an adaptive response to increased energy demand, marked by coordinated cardiomyocyte growth in length and width, without adverse remodeling [27,31,66]. Swimming, running on a treadmill, and voluntary wheel activities have been shown to induce physiological hypertrophy and cardiomyocyte proliferation [27,66,67]. As improved VO_2_max is a crucial determinant of myocardial protection, HIIT, which is well suited for VO_2_max enhancement, emerges as a potent non-pharmacological conditioning strategy [68,69]. Additionally, aerobic exercise can increase left ventricular systolic pressure [70], further supporting its role in enhancing cardiac performance under stress. Therefore, we suggest that the benefits of HIIT in this model may be largely attributed to its anti-inflammatory effects, as evidenced by the significant reductions in inflammatory cytokines. In contrast, only partial changes were observed in apoptosis-related markers, leaving the role of anti-apoptotic mechanisms uncertain. Other potential mechanisms, such as alterations in cardiac metabolism, contractile function, or physiological hypertrophy, remain speculative and require further investigation. It is well established that the PI3K/AKT/mTOR signaling pathway directly regulates several apoptosis-related proteins, including Bcl-2, caspase-3, and Bax, thereby influencing cell survival, metabolism, growth, and proliferation [71,72]. Studies on cardiac tissue and isolated cardiomyocytes have further demonstrated that insulin-like growth factor I (IGF-I) or exercise activates PI3K, subsequently triggering Akt and its downstream targets to regulate physiological increases in cell size and cardiac hypertrophy [73,74]. In addition, findings from rat models of ischemia–reperfusion (IR) injury indicate that high-intensity interval training (HIIT) downregulates the vascular inhibitory factor thrombospondin-1 (TSP-1) via the PI3K/Akt pathway, thereby modulating apoptosis through anti-apoptotic mediators such as BCL-2, survivin, and XIAP, accompanied by increased nitric oxide levels [75,76]. Collectively, these studies highlight the critical role of PI3K/Akt signaling in mediating cardioprotective mechanisms. In our study, HIIT significantly reduced activated caspase-3 and TNF-α, while Bax showed a modest non-significant downward trend. TUNEL staining did not show a significant change in myocardial apoptosis, suggesting modulation of apoptosis- and inflammation-related signaling rather than a decrease in myocardial apoptosis. These patterns suggest that the observed effects of HIIT may be associated with PI3K/Akt-related signaling pathways involved in inflammatory regulation. However, as endothelial and vascular factors were not directly assessed in the present study, this potential mechanism remains speculative and warrants further investigation. Beyond these potential PI3K/Akt-related effects, our study confirmed that AKI induced systemic inflammation, reflected by elevated TNF-α, IL-1β, and IL-6 levels and reduced IL-10. Western blot analysis revealed increased caspase-3, TNF-α, and Bax expression in myocardial tissues, consistent with apoptosis and inflammation. HIIT preconditioning significantly reduced inflammatory cytokines and showed partial reductions in apoptosis-related markers, indicating that anti-inflammatory mechanisms and possibly limited anti-apoptotic effects may contribute to the observed improvements. Similar findings have been reported in exercise preconditioning studies, such as swimming prior to myocardial infarction, which improved antioxidant capacity and contractile function [77,78]. Clinical evidence also shows that HIIT is superior to moderate-intensity continuous training in improving endothelial function [64,79,80,81,82]. This benefit is attributed to exercise-induced increases in heart rate and blood flow, which generate shear stress in vascular endothelium, activating endothelial nitric oxide synthase (eNOS) and enhancing nitric oxide (NO) production [80,81,82,83]. NO is a central regulator of vascular homeostasis, and exercise-induced eNOS activation reduces oxidative stress and calcium overload during renal I/R, protecting cardiomyocytes from death [84,85]. Evidence further suggests that exercise may preferentially activate eNOS in the coronary artery endothelium, enhancing vasodilation and conferring protection against I/R injury [86]. Aerobic exercise reduces lipids, glucose, thrombosis, and inflammation in patients with metabolic and ischemic diseases, thereby improving prognosis [87]. Although prior studies suggest that the eNOS/NO pathway may contribute to exercise-induced cardioprotection, this mechanistic explanation remains speculative in the present study, as we did not directly measure eNOS expression or NO production. Taken together, the literature supports a potential role for eNOS/NO signaling in exercise-related vascular adaptations. However, in the present study, the observed benefits of HIIT were mainly consistent with attenuation of inflammatory responses, and the contribution of eNOS/NO signaling remains undetermined because it was not directly assessed. This hypothesis provides a promising direction for future mechanistic studies.

Despite our promising results, this study had several limitations that should be considered. First, this study did not include an HIIT-only control group without renal I/R, which represents an important limitation. Because all trained rats were required to undergo renal I/R to evaluate exercise-induced preconditioning, a separate HIIT-only group was not incorporated into the original study design. Therefore, we cannot entirely exclude the possibility that HIIT may have induced baseline cardiac or renal adaptations, such as physiological hypertrophy, changes in inflammatory status, or alterations in renal hemodynamics, which could influence the magnitude of protection observed after renal I/R. This limitation introduces uncertainty as to whether the observed cardiac responses were solely attributable to attenuation of renal I/R injury or were partly influenced by exercise-induced baseline adaptations. Therefore, future studies should include a dedicated HIIT-only group to clarify these baseline effects and determine how they interact with subsequent renal I/R injury. Second, although the study was initially designed with 10 animals per group, perioperative mortality inherent to the renal I/R model resulted in only six analyzable subjects in some groups. This reduction may have limited statistical power; however, the use of non-parametric statistical methods and consistent effect trends across multiple endpoints partially offset this limitation. Third, although this study evaluated multiple biochemical, histological, and apoptosis-related outcomes, we did not investigate the upstream molecular signaling pathways that may underlie these changes. Previous research has implicated mitochondrial apoptotic regulators, oxidative stress-related MAPK activation, and NF-κB-mediated inflammatory signaling; however, these pathways were not measured in the present study. Therefore, our mechanistic interpretations, including the potential involvement of the eNOS/NO pathway, should be considered speculative. Future studies should include targeted molecular analyses to validate the upstream signaling mechanisms responsible for the observed inflammatory modulation induced by HIIT following renal I/R. Finally, future studies with larger sample sizes and inclusion of an exercise-only group will be required to further confirm and extend these findings.

## 5. Conclusions

In this study, we established a rat model of acute kidney injury induced by renal ischemia–reperfusion, which resulted in clear biochemical and histological evidence of secondary myocardial injury. Four weeks of HIIT preconditioning provided partial cardioprotective and renoprotective effects, as reflected by significant reductions in BUN, creatinine, tubular injury scores, cardiac troponin I, pro-inflammatory cytokines, and myocardial caspase-3 and TNF-α expression. Other parameters, including Bax expression, myocardial morphology, and TUNEL-positive cells, showed modest but non-significant trends toward improvement, suggesting that the protective effect of HIIT may be selective rather than global across all measured outcomes.

These patterns are consistent with previous research, indicating that exercise may attenuate inflammation and apoptosis during ischemia–reperfusion injury. Although the present study did not directly assess upstream signaling pathways, the observed reductions in apoptosis-related markers are compatible with mechanisms previously linked to PI3K/Akt signaling and nitric oxide-mediated endothelial protection; however, these interpretations remain speculative and warrant further investigation.

From a translational perspective, structured exercise training remains a practical, accessible, and low-cost strategy that may help improve cardiovascular resilience in individuals at risk of AKI. Future studies incorporating larger sample sizes, mechanistic analyses, and an HIIT-only control group are necessary to clarify the extent and mechanisms of HIIT-induced protection in the setting of renal I/R.

## Figures and Tables

**Table 1:** Overview of the high-intensity interval training protocol.

Stage (Week)	Speed (m/min)	Incline (°)	Duration (min)	Interval	Frequency (Bouts/Week)
3	20	5	30	2 min rest + 1 min sprint × 10	4
4	30	10	30		
5–8	50	10	30		

**Table 2:** Mortality rates.

Group	Sham	Renal Ischemia/Reperfusion	High-Intensity Interval Training
Rats allocated	10	10	10
Deaths recorded	0	1	2
Final sample size	10	9	8

**Table 3:** Hemodynamic changes during the experiments.

	Systolic Blood Pressure, mmHg			Heart Rate, Beats/min		
Group	Sham	Renal (I/R)	HIIT	Sham	Renal (I/R)	HIIT
Baseline 1	105 ± 4	104 ± 3	102 ± 5	457 ± 15	454 ± 22	454 ± 28
Baseline 2	104 ± 3	106 ± 5	104 ± 4	459 ± 21	458 ± 27	455 ± 23
Renal artery occlusion (RAO) (1 h)	102 ± 5	77 ± 8	82 ± 6	451 ± 20	373 ± 31	387 ± 25
RAR (3 h)	103 ± 4	86 ± 7	91 ± 9	455 ± 26	385 ± 26	396 ± 29
RAR (24 h)	105 ± 3	68 ± 7	74 ± 5	454 ± 18	342 ± 30	371 ± 36

Baseline 1 = pre-abdominal surgery; Baseline 2 = post-abdominal surgery; RAO (1 h) = renal artery occlusion after 1 h; RAR (3 h) = renal artery reperfusion after 3 h. I/R, ischemia–reperfusion; HIIT, high-intensity interval training.

## Data Availability

The data presented in this study are available on request from the corresponding author. The data are not publicly available due to ethical and institutional restrictions.

## Supplementary Materials

The following supporting information can be downloaded at: https://www.mdpi.com/article/10.3390/life16010044/s1, Figure S1: Representative image of rats running on a treadmill during the HIIT protocol. The dark metallic panel at the rear of the treadmill is the mild stimulus grid, which provides a minimal behavioral cue only when a rat drifts toward the back of the running lane; Figure S2: Representative appearance of rat kidneys during ischemia. The left panel shows a normally perfused kidney before vascular clamping, displaying a pale reddish coloration, whereas the right panel shows the kidney after 1 h of ischemia, appearing darker with evident congestion. Figure S3: Experimental procedure during the 60 min ischemia period. After both renal pedicles were clamped, the intestines were gently repositioned and covered with sterile gauze, and the kidneys remained ischemic for 60 min. The abdominal cavity was kept open but protected throughout the period to maintain surgical stability and prevent contamination.

## Abbreviations

The following abbreviations are used in this manuscript:

AKI	Acute kidney injury
BUN	blood urea nitrogen
CPK	creatine phosphokinase
cTnI	cardiac troponin I
ELISA	enzyme-linked Immunosorbent
eNOS	endothelial nitric oxide synthase
HIIT	high-intensity interval training
IL	interleukin
I/R	ischemia–reperfusion
LDH	lactate dehydrogenase
NO	nitric oxide
TNF-α	tumor necrosis factor alpha
TUNEL	Transferase dUTP Nick-end Labeling
