# Supplementary figures and images for: High-Intensity Interval Training Attenuates Inflammation in Cardiorenal Syndrome Induced by Renal Ischemia–Reperfusion Injury in Rats
Source: Life (Basel). 2025 Dec 26;16(1):44. doi: 10.3390/life16010044 (PMC12843421; doi:10.3390/life16010044)

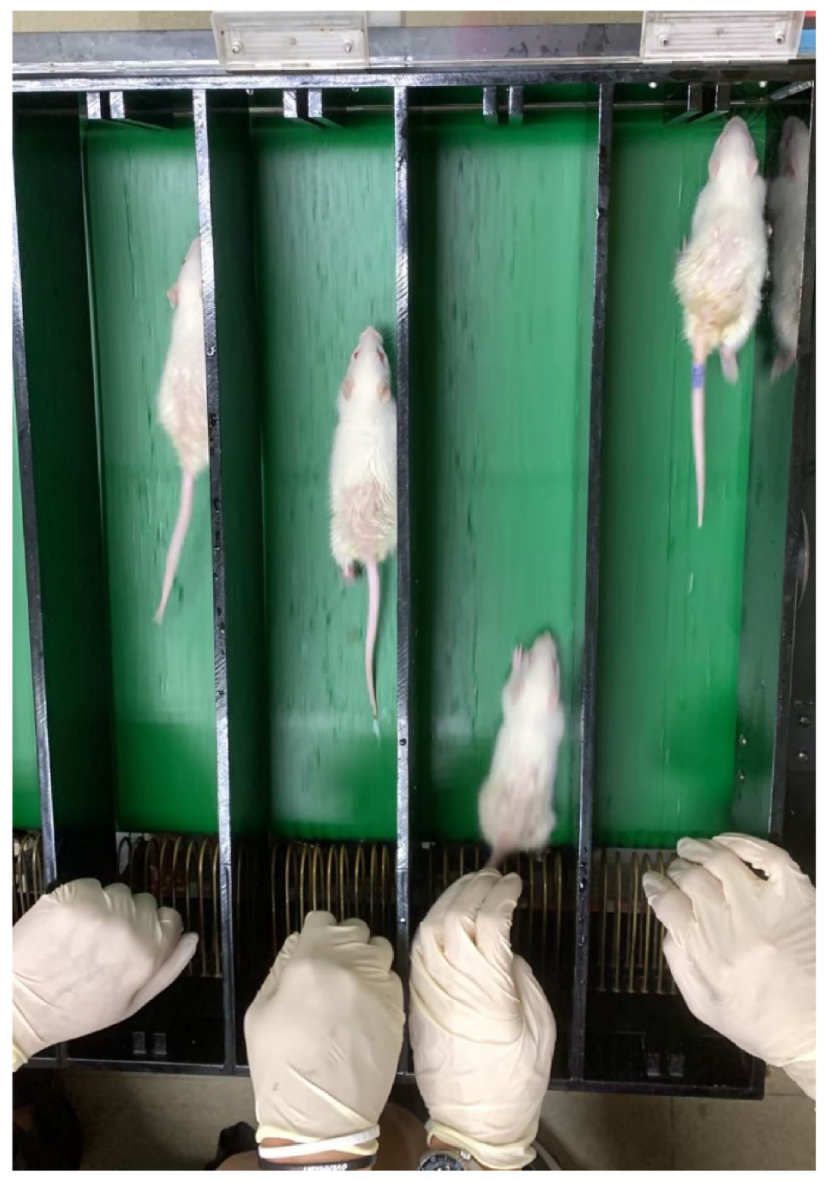

Supplement: Supplementary file 1 [file life-16-00044-s001.zip › Figure S1.tif]

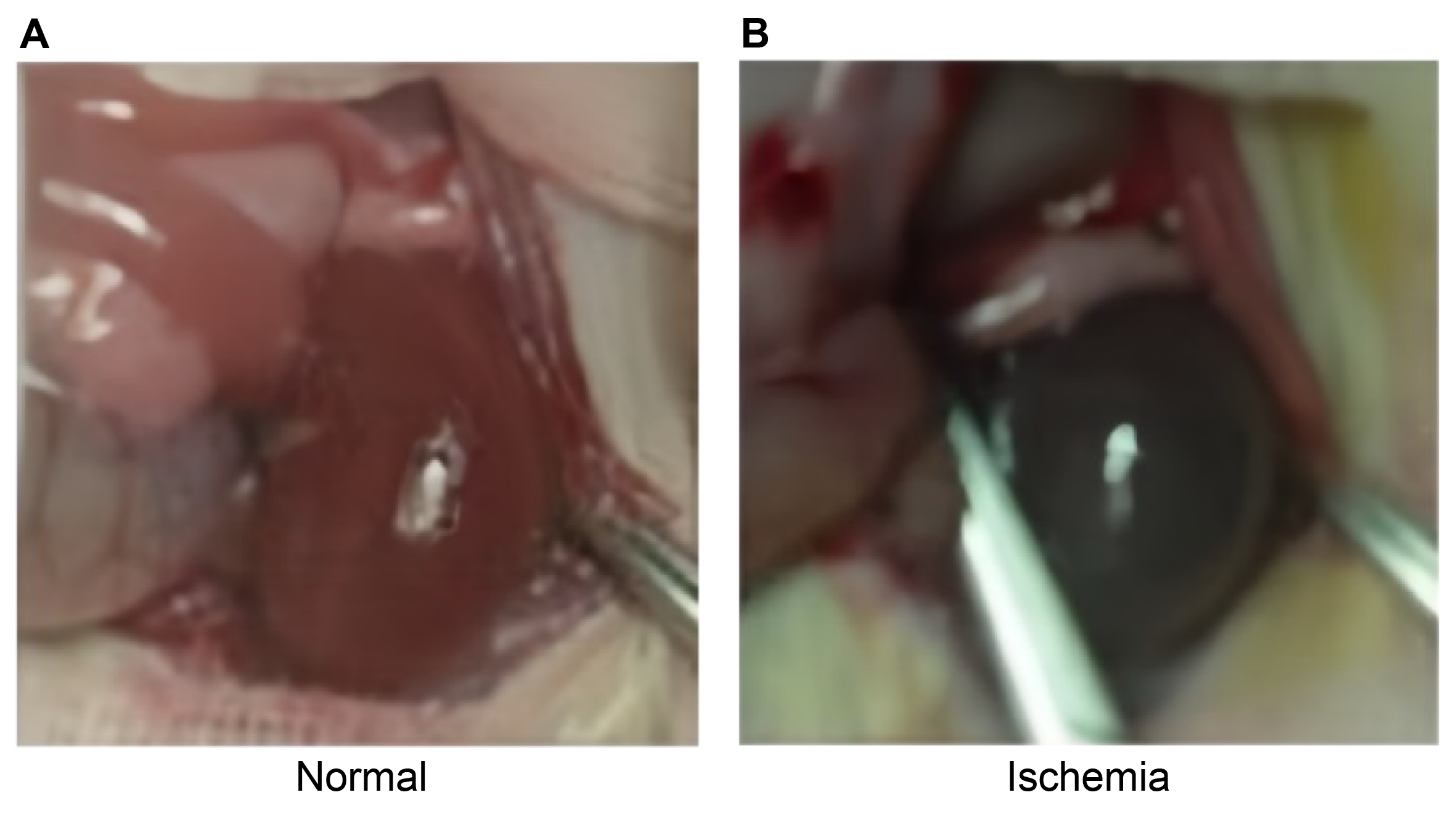

Supplement: Supplementary file 1 [file life-16-00044-s001.zip › Figure S2.tif]

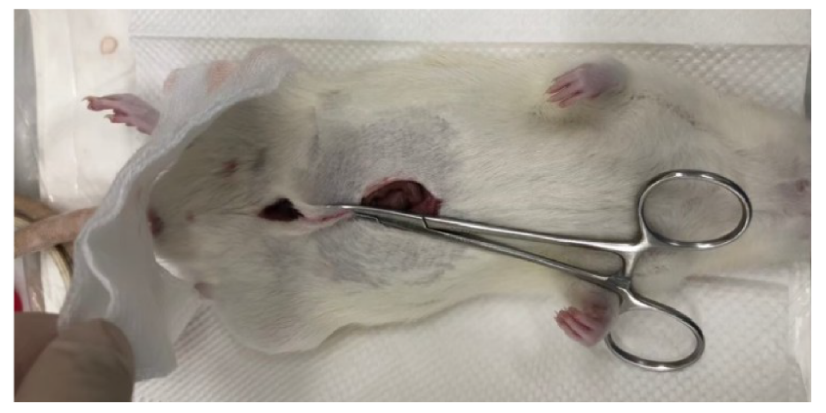

Supplement: Supplementary file 1 [file life-16-00044-s001.zip › Figure S3.tif]
